# Investigating Balloon-Vessel Contact Pressure Patterns in Angioplasty: In Silico Insights for Drug-Coated Balloons

**DOI:** 10.1007/s10439-023-03359-y

**Published:** 2023-09-26

**Authors:** Efstathios Stratakos, Luca Antonini, Gianluca Poletti, Francesca Berti, Abraham R. Tzafriri, Lorenza Petrini, Giancarlo Pennati

**Affiliations:** 1 Laboratory of Biological Structure Mechanics, Department of Chemistry, Materials and Chemical Engineering “Giulio Natta”, Politecnico di Milano, Milan, Italy; 2 CBSET Inc, Lexington, MA USA; 3 Department of Civil and Environmental Engineering, Politecnico di Milano, Milan, Italy

**Keywords:** Drug-coated balloons, Contact pressure, Non-uniformity, Finite element simulations, Numerical, Drug-coating

## Abstract

Drug-Coated Balloons have shown promising results as a minimally invasive approach to treat stenotic arteries, but recent animal studies have revealed limited, non-uniform coating transfer onto the arterial lumen. In vitro data suggested that local coating transfer tracks the local Contact Pressure (CP) between the balloon and the endothelium. Therefore, this work aimed to investigate in silico how different interventional and device parameters may affect the spatial distribution of CP during the inflation of an angioplasty balloon within idealized vessels that resemble healthy femoral arteries in size and compliance. An angioplasty balloon computational model was developed, considering longitudinal non-uniform wall thickness, due to its forming process, and the folding procedure of the balloon. To identify the conditions leading to non-uniform CP, sensitivity finite element analyses were performed comparing different values for balloon working length, longitudinally varying wall thickness, friction coefficient on the balloon-vessel interface, vessel wall stiffness and thickness, and balloon-to-vessel diameter ratio. Findings indicate a significant irregularity of contact between the balloon and the vessel, mainly affected by the balloon’s unfolding and longitudinal thickness variation. Mirroring published data on coating transfer distribution in animal studies, the interfacial CP distribution was maximal at the middle of the balloon treatment site, while exhibiting a circumferential pattern of linear peaks as a consequence of the particular balloon-vessel interaction during unfolding. A high ratio of balloon-to-vessel diameter, higher vessel stiffness, and thickness was found to increase significantly the amplitude and spatial distribution of the CP, while a higher friction coefficient at the balloon-to-vessel interface further exacerbated the non-uniformity of CP. Evaluation of balloon design effects revealed that the thicker tapered part caused CP reduction in the areas that interacted with the extremities of the balloon, whereas total length only weakly impacted the CP. Taken together, this study offers a deeper understanding of the factors influencing the irregularity of balloon-tissue contact, a key step toward uniformity in drug-coating transfer and potential clinical effectiveness.

**Supplementary Information:**

The online version contains supplementary material available at 10.1007/s10439-023-03359-y.

## Introduction

Cardiovascular diseases are the primary cause of death globally, with heart attack and stroke contributing to 85% of all cardiovascular diseases’ deaths in 2019 [11]. Atherosclerosis, which is a pathological condition affecting the intimal layer of large and medium-sized arteries, is the leading cause of cardiovascular diseases. This condition causes the formation of atheromatous, also known as atherosclerotic plaques, resulting in a significant increase in the thickness of the arterial wall and in arterial stenosis. Peripheral artery disease is a specific subset of cardiovascular diseases that primarily affects the blood vessels supplying the arms and legs. It is a widespread condition, impacting over 230 million individuals globally [16].

Drug-coated balloons (DCBs) have emerged as a promising, minimally invasive therapeutic intervention for the treatment of arterial stenosis and have shown promising results in the treatment of peripheral artery disease. During the treatment, antiproliferative drug particles are delivered to the media and adventitia layers of the arterial wall to foster the inhibition of smooth muscle cell proliferation. The drug is encapsulated within an excipient layer that is designed to be transferred and remain attached to the arterial endoluminal surface of the diseased vessel, acting as a long-term drug depot for the lesion area [9]. Therefore, DCBs offer the potential advantages of (i) promoting the “leave-nothing-behind” strategy and (ii) uniformly delivering drug-coating on the lesion, due to their morphological structure that facilitates complete contact with the stenosed vessel.

Although some clinical cohorts have shown promising results, a clinical study revealed that the efficacy of the treatment can be limited by calcification [20]. However, the exact mechanism by which calcification limits treatment efficacy is not yet fully understood. It remains unclear whether the limitations arise from apposition restrictions or hindrance of drug absorption and diffusion by calcific barriers [55]. The coating on DCBs may undergo significant loss en-route to the target area. This may result in incomplete coverage of the balloon surface prior to its interaction with the target area [19, 29], with the drug loss during tracking reaching up to 20–30%, in vitro [34] and 18–30% in pigs. Once inflated in the lesion, the interaction of the DCB with the arterial wall will determine the efficacy of local coating delivery. Notably, a recent study using healthy pigs revealed sparse coating adherence to the luminal wall of femoral arteries 30 min post intervention with clinical grade DCB, in a manner that was not explained by coating loss during tracking [56]. More specifically, linearly patterned distributions of coating transfer were evident, with reduced values at the two ends. Such findings indicate that there is potential for further optimization of the treatment, primarily by refining the device itself. To this end, the chemical optimization of the drug-coating formulation is the usual focus in the development of DCBs [1, 35, 38, 54].

However, as highlighted in a recent review, the biophysical forces affecting coating transfer have received less attention [51]. Efforts to determine the biophysical parameters driving the transfer of the coating to the arterial mural surface identified the Contact Pressure (CP) between the external surface of the balloon and the endoluminal surface as a heavy-impact parameter [10]. Increased CP has been shown to enhance the transfer and penetration depth of the coating into the vessel [12, 56], which is likely to result in a higher amount of drug being delivered to the vascular tissue [49]. Consistently, a micro-patterned DCB demonstrated that the higher local CP results in elevated drug delivery and subsequent improvement in vessel patency [39]. In previous works, Chang et al. and Tzafriri et al. presented the concept of micromechanical indentation pressure, developed during the interaction of the coating with the vessel, as the responsible cause for the coating transfer [12, 56]. Notably, this is driven by the macro-mechanical CP developed during the interaction of the DCB with the vessel. However, due to the infeasibility or complexity of the measurement, so far neither in vivo human or animal studies nor in vitro analyses have assessed CP during treatment. Moreover, in silico simulations, that have been widely adopted to study angioplasty procedures, have not yet been utilized to specifically estimate the arising CP distribution during the balloon-artery interaction.

Therefore, in this study, we were interested in exploiting finite element simulations to investigate the interaction between angioplasty balloons and simplified vessels that resemble femoral arteries, to compare the findings with previous animal experiments on coating transfer [56].

In this effort, it is critical to adopt a suitable balloon numerical model able to capture the details of the contact modalities. Over the past 20 years, various models have been developed in literature to simulate the mechanical behavior of angioplasty balloons. These models range from simple to complex approaches of high stiffness cylinders [28, 53, 58, 60], compliant cylinders [30, 33, 36], axis-symmetric models [56], multi-winged models [3, 6, 14, 45], reconstructed geometries [7, 8, 27, 37, 43, 44, 57, 61] and distended models that incorporated the folding process [4, 17, 26, 59]. The latter was adopted in the current work as the most accurate and most suitable to study the interaction between the balloon and the vessel. In parallel, evidence in the literature suggests that angioplasty balloon catheters exhibit a non-uniform thickness along the longitudinal axis of the balloon [22], while the tapered area is expected to have a significantly higher thickness, attributed to their blow molding manufacturing process. Therefore, varying wall thicknesses along the longitudinal axis of the balloon were likewise considered in the balloon numerical model.

Given the factors previously mentioned, the current study aimed to examine the potential of contact non-uniformity arising during balloon inflation, attributed to the balloon folding and longitudinal thickness variation. To this end, the balloon interaction with idealized vessels were considered, in order to avoid the introduction of other sources of contact non-uniformity (e.g., due to asymmetric lumen or lesion complexity as in human atherosclerotic arteries). Due to parameters variability, a sensitivity analysis was performed to study the impact of various procedural conditions on the balloon-vessel interaction. To investigate these, numerical models of a generic peripheral balloon catheter, incorporating its folding process, were adopted and expanded within idealized vessels, straight and cylindrical, resembling healthy femoral arteries in terms of diameter, wall thickness, and compliance. The models were used to generate CP maps in different conditions, considering various balloon and vessel characteristics, including (i) the balloon working length, (ii) the balloon’s longitudinal wall thickness variation, (iii) the vessel wall stiffness, (iv) the vessel wall thickness, (v) the friction coefficient on the balloon-vessel interface, and (vi) the balloon-to-vessel diameter ratio.

## Materials and Methods

### Balloon Model

**Fig. 1:**
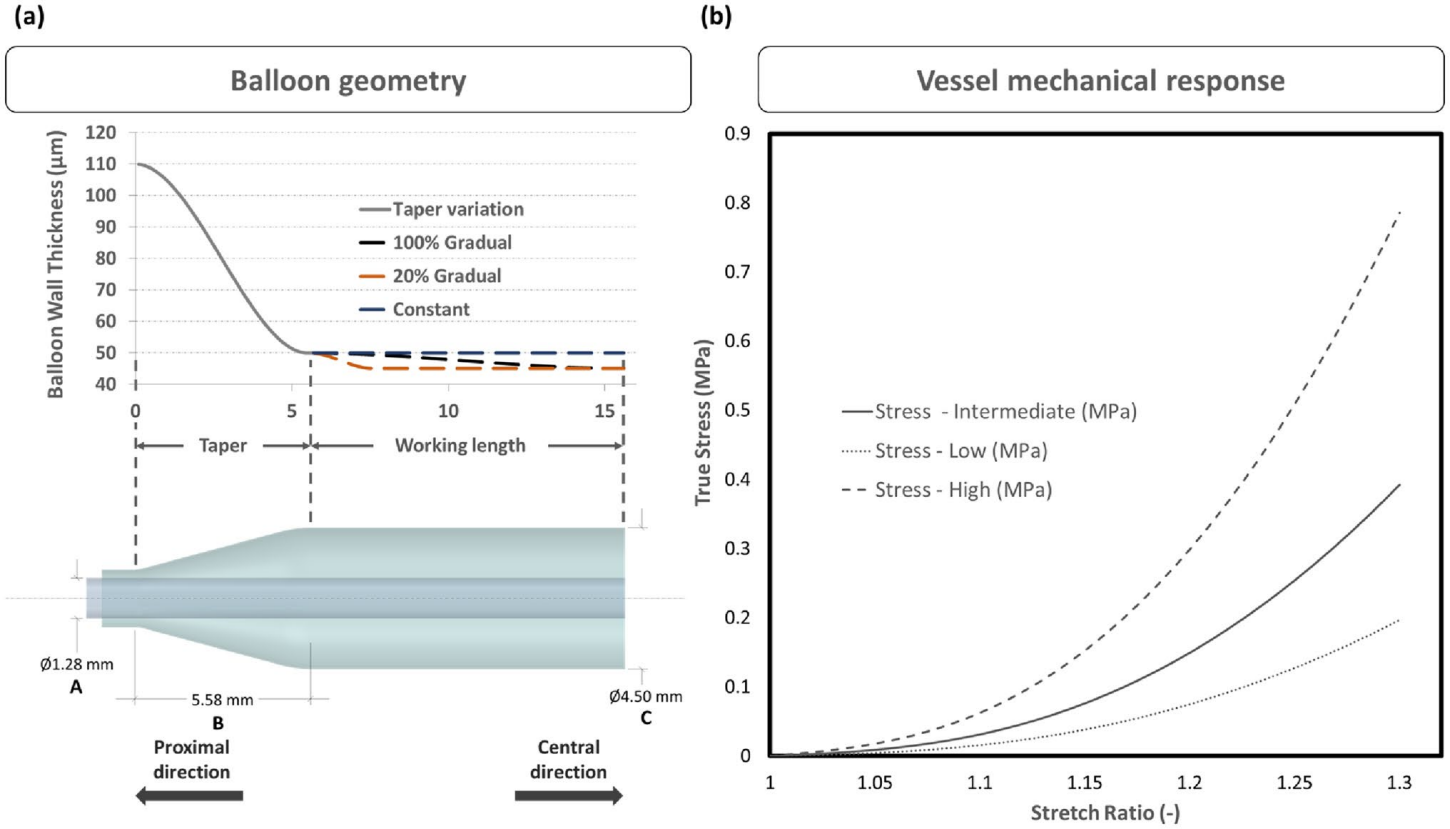
**a** Bottom—Dimensions of the angioplasty balloon model: “A” refers to the diameter of the catheter, “B” indicates the length of the taper on the balloon, and “C” represents the balloon diameter along the working length; the working length of the catheter was modified based on various values outlined in Table 1. Top—the three considered thickness variation cases. **b** Mechanical response of the three considered vessel material models

Angioplasty balloons can be categorized into three types according to their radial compliance: compliant, semi-compliant, and non-compliant. Drug-coated balloons (DCBs) typically fall under the semi-compliant category, which means these balloons can expand by up to 30% of their original diameter when they reach their rated burst pressure. In this work, a standard semi-compliant balloon catheter, which is the usual substrate for DCB catheters [15], was employed as the balloon model (Fig. 1a). Because of the symmetry in the numerical problem, half-symmetry conditions were utilized in all the simulations and deemed to be an appropriate balance between computational efficiency and accuracy. To investigate the effect of working length on balloon performance, three different cases were considered with working lengths of 10 mm, 20 mm, and 50 mm (referring to the half-symmetry balloon model). To vary the thickness of the balloon taper, a gradient was applied along its length, which decreased from 110 μm at the proximal part to 50 μm at the end of the taper according to a smooth step function (Fig. 1a). Considering the uncertainty of the wall thickness variation across the working length of the balloon, 3 different wall thickness cases were modeled: (i) 100% Gradual: a linear decrease from 50 μm at the proximal end of the working length to 45 μm at the central end, (ii) 20% Gradual: a linear decrease from 50 μm at the proximal end of the working length to 45 μm over the 20% of the working length toward the central direction, followed by a constant thickness of 45 μm for the remaining length, and (iii) Constant: a constant thickness of 50 μm (Fig. 1a).

Pebax materials are typically used for semi-compliant balloons. These materials exhibit a linear and isotropic behavior for stretch up to 25%, demonstrating also a softer behavior compared to Nylon, another material frequently adopted for angioplasty balloons (about 500 MPa vs about 1.1 GPa [40, 48]). Nevertheless, to the best of authors’ knowledge, no direct measurements are available for the mechanical properties of the balloon after the folding process: as blades of the folding and pleating devices are heated, some changes in the material properties are likely to occur [26]. An alternative, simplified, approach to deduce reasonable mechanical properties for the commercial balloons is to rely on the compliance charts (pressure-diameter relationship for a range of pressures) declared by the producers [2, 13, 42, 62]. For the sake of simplification, the balloon material was modeled as homogeneous, isotropic linear elastic material model, an approach widely adopted in the literature [2, 8, 26, 41]. A Young's modulus of 400 MPa was assumed, which produces a mechanical response of the balloon models (for the three investigated wall thickness cases) during a free expansion simulation (Fig. 3), consistent with the compliance chart of a commercial peripheral DCB (https://www.l2mtech.de/?page_id=462). Moreover, in a recent numerical study comparing Nylon and Pebax balloons, a Young’s modulus of 414 MPa was adopted to mechanically describe the performance of the Pebax [40], which is in line with the current material model. Finally, a Poisson’s ratio of ν  = 0.4 and a density of *ρ* = 916 kg/m^3^ are here used for the balloon’s material [26].

**Fig. 2:**
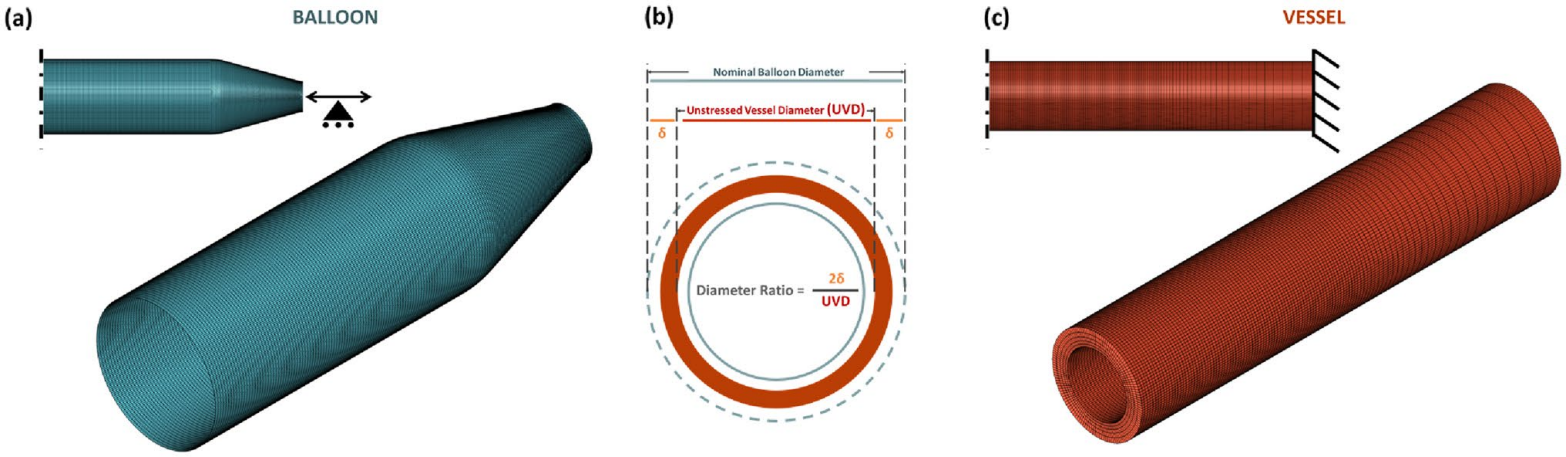
**a** Mesh and boundary conditions adopted for the balloon numerical model, **b** Diagrammatic explanation of the Diameter Ratio, and **c** Mesh and boundary conditions adopted for the vessel numerical model

Shell elements, as adept at capturing the inter-elemental bending stiffness, were used in the numerical representation of the balloon to simulate the balloon's response to the highly bending conditions introduced during the folding process. In particular, reduced integration linear elements were adopted: the element distribution was carefully analyzed, and a mesh sensitivity study was performed on the control case simulation of balloon inflation in a vessel (Supplemental Fig. 1). By increasing the number of elements on the circumference and using a finer mesh in the tapered region, the curve inner pressure-maximum diameter and the CP showed minimal variation, when the number of elements exceeded 300 circumferentially. Adopting the aforementioned meshing approach, the 10-, 20-, and 50-mm balloons were each discretized using a total mesh of 51000, 81000, and 171600 four-noded, reduced integration, isoparametric elements, respectively (Fig. 2a).

### Vessel Model

As in animal studies [56], idealized cylinders mimicking the geometry of healthy femoral arterial wall were used and a single homogeneous material was adopted to replicate the whole vessel wall. The thickness of the vessel walls was chosen to be consistent with measurements taken from human femoral arteries at 1.1 mm [5] and was then varied by ± 550 μm to account for variability. Moreover, a diameter ratio (DR) was here defined as the measure of the relationship between the nominal diameter of the balloon (the diameter at nominal pressure) and the lumen diameter of a vessel and is expressed as a percentage ratio ((balloon diameter − vessel diameter)/vessel diameter) (Fig. 2b). The inner diameter of the vessel model was varied from 3.55 to 4.25 mm, to match different DR conditions (Table 1). The considered vessel length was 15 mm longer than the overall length of the balloon model in each case, so that the balloon was fully contained within the vessel, preventing any interference from the ends of the vessel on the balloon's expansion.

**Table 1:** Conditions and variables adopted in the simulations

Conditions	Value 1 (control)	Value 2	Value 3	Value 4
Device parameters				
Working length (mm)	10	20	50	***–***
Friction coefficient	0.2	0	0.5	***–***
Thickness variation	100% gradual	20% gradual	Constant	***–***
Procedural parameters				
Diameter ratio (%)	18	25	32	40
Vessel stiffness	Intermediate	Low	High	*–*
Vessel wall thickness (μm)	1100	550	1650	–

The vessel mechanical behavior was described using the 3rd-order Mooney Rivlin hyperelastic constitutive model and the parameters were selected to imitate femoral arteries, as suggested by Prendergast et al. [46]. The model was set as nearly incompressible, which implies using a Poisson's ratio of 0.475 (default option for the used numerical code). To account for tissue variability, three different stress-stretch curves were considered covering a range (Fig. 1b) consistent with literature data on peripheral arteries [32, 46].

The vessel geometries were discretized using a mesh comprising from 69,960 to 201,300 three-dimensional, eight-noded, reduced integration, linear hexahedral elements, depending on the length of the vessel. Five elements were imposed radially, decreasing in size toward the lumen. Increased element density was also adopted in the longitudinal area of balloon-vessel interaction. A set of nodes was encastered on the proximal end of the vessel, while symmetry conditions were considered in the central extremity (Fig. 2c).

### Simulations

The performed numerical simulations included the following: (i) the balloon’s folding process to acquire its folded configuration, (ii) the balloon’s free expansion to describe its unfolding process and compliance, and (iii) the balloon’s inflation in idealized vessels to investigate the balloon-vessel interaction. The balloon folded geometry and its unfolding process determine the initial contact with the vessel, hence in this work particular attention was devoted to simulating the two steps. The folding process of the catheter as described by Ref. [26] was employed, using 3 rigid folding jaws and 10 rigid pleating plates. The boundary conditions adopted for the symmetric balloon model were consistent with with Ref. [26, 59] and are demonstrated in Fig. 2a, while the boundary conditions of the artery can be seen in Fig. 2c. The balloon folding and unfolding simulations involve a multitude of nonlinearities due to high levels of deformation, multiple points of contact, and nonlinear material behavior. During the folding process, the effects of inertia are typically deemed insignificant. However, during the unfolding of a balloon, the dynamic effects are highly dependent on the rate at which the balloon is inflated. Previously, numerical simulations of balloon expansion have typically utilized either a static or quasi-static framework, utilizing either implicit [26] or explicit [4, 58, 60] solvers, respectively. While implicit solvers might be set to completely neglect inertial effects, explicit solvers are only able to approximate quasi-static behavior when the kinetic-to-internal energy ratio is kept below 5–10% for the majority of the expansion process. Considering the above, the authors adopted a dynamic explicit solution scheme using the Abaqus/Explicit solver (Dassault Systemes Simulia Corp, Johnston RI). Both during the free expansion of the balloon and during the inflation within the vessels, adequate analysis time was implemented, so that the ratio of kinetic energy to internal energy remained below 5% for the majority of the unfolding simulation. During the simulations, viscous pressure was applied to both the inner and outer surfaces of the vessels and the balloon to mitigate oscillations and dynamic effects and to enhance the stability of the simulation. To characterize the interaction between the different contact pairs, a general contact formulation was adopted which uses sophisticated tracking algorithms to ensure that proper contact conditions are enforced efficiently. “Hard” contact was used for the Pressure-Overclosure relationship to describe the normal behavior and different friction coefficients (ranging from 0.0 to 0.5) were considered to describe the tangential behavior. A friction coefficient of 0.2 was adopted both for the contact between the balloon’s internal surface and the inner catheter and for the self-contact of the internal and external surfaces of the balloon, which is consistent with previous studies [4, 41, 43, 57, 59].

For the contact between the vessel lumen wall and the external surface of the balloon, different values of the friction coefficient were tested (from 0 to 0.5, assuming 0.2 for the control case). The pressure variations on the internal surface of the balloon were modeled using a "smooth step" function to minimize abrupt transitions and minimize dynamic effects. The pressure initiated at a value of 0 atm, decreased to − 1 atm during the folding process and then increased until a maximum value of 13 atm during the expansion.

A suite of numerical simulations was conducted to evaluate the impact of various procedural and modeling factors on the modalities of balloon-vessel interaction (Table 1). A baseline case, referred to as the control case, was established and its parameters were used as the reference point for all subsequent analyses. The intention was to evaluate the impact of the variability of device and procedural parameters on a particular outcome. To achieve this, all parameters of the control case were maintained except for one, which was systematically altered to determine its individual effect on the outcome.

### Post-processing

A script was developed utilizing the programming language MATLAB to facilitate the post-processing of data generated from this study examining the pressure distribution along the cylindrical vessel structure. This script was designed to receive as input the CP values determined at the nodes of the mesh along the vessel structure. The location of each point was specified using a cylindrical coordinate system defined by the longitudinal center axis of the vessel as the *z*-axis, and the circumferential and radial axis as the *θ* and r axis, respectively. The script produces a map that utilizes a color code to depict the intensity of the stress at each point. The pixel number of the map corresponded to the number of nodes along the vessel endoluminal surface.

## Results

**Fig. 3:**
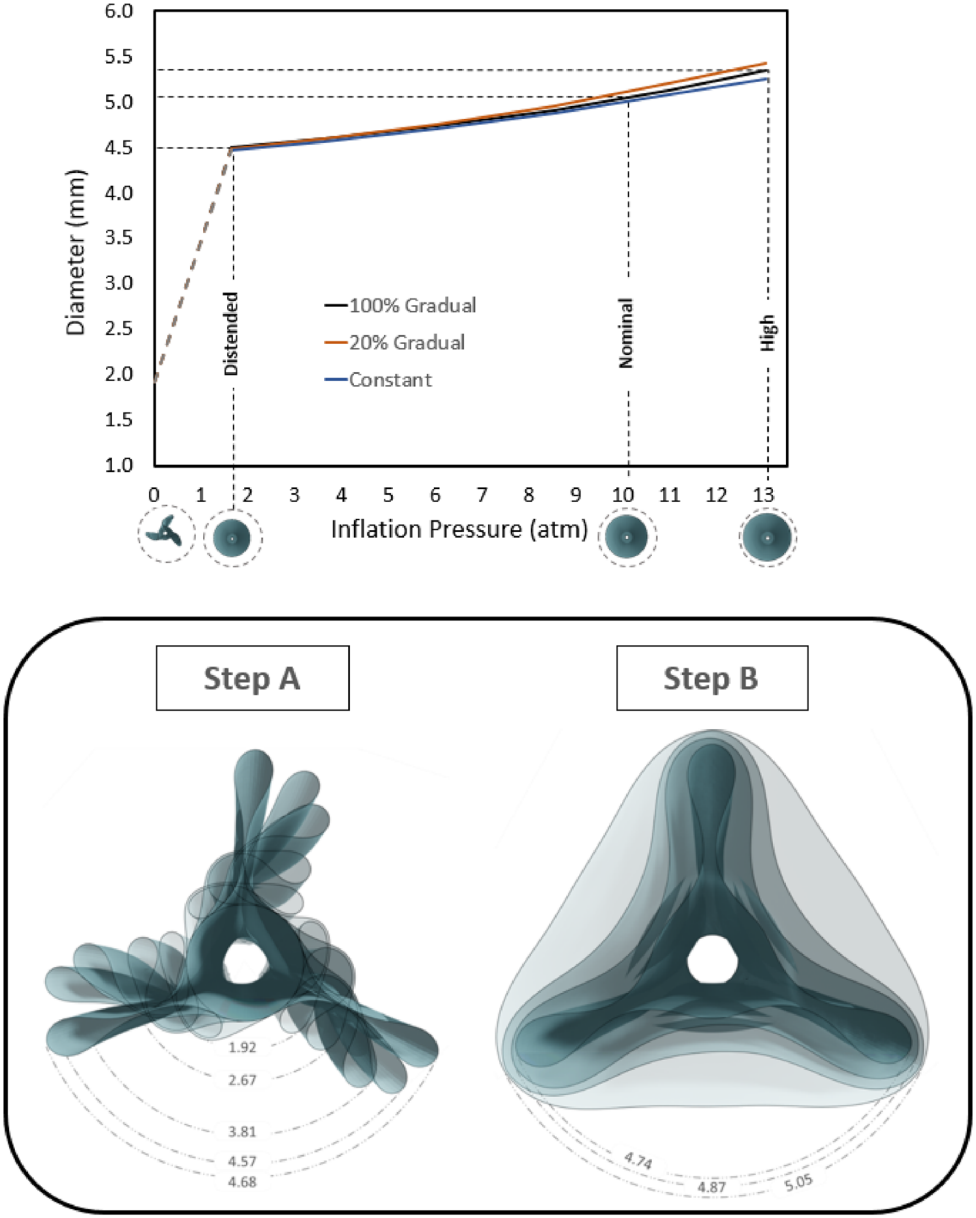
Top: Relationship between the inflation pressure and the diameter (only after balloon distension) during a free expansion for the three balloon models with different thickness variations; Bottom: balloon behavior before its complete distention (Inflation pressure from 0 to 1.6 atm). The different unfolding configurations and the maximum distance from the axis are indicated. Step A reveals the initial unfolding movement during which the folds rotate around the central axis of the balloon. Subsequently, in step B, the balloon will start to distend until a circular cross-section is reached, where the balloon will start to increase the diameter of the cross-section. The values vary insignificantly with the different thickness variation conditions

**Fig. 4:**
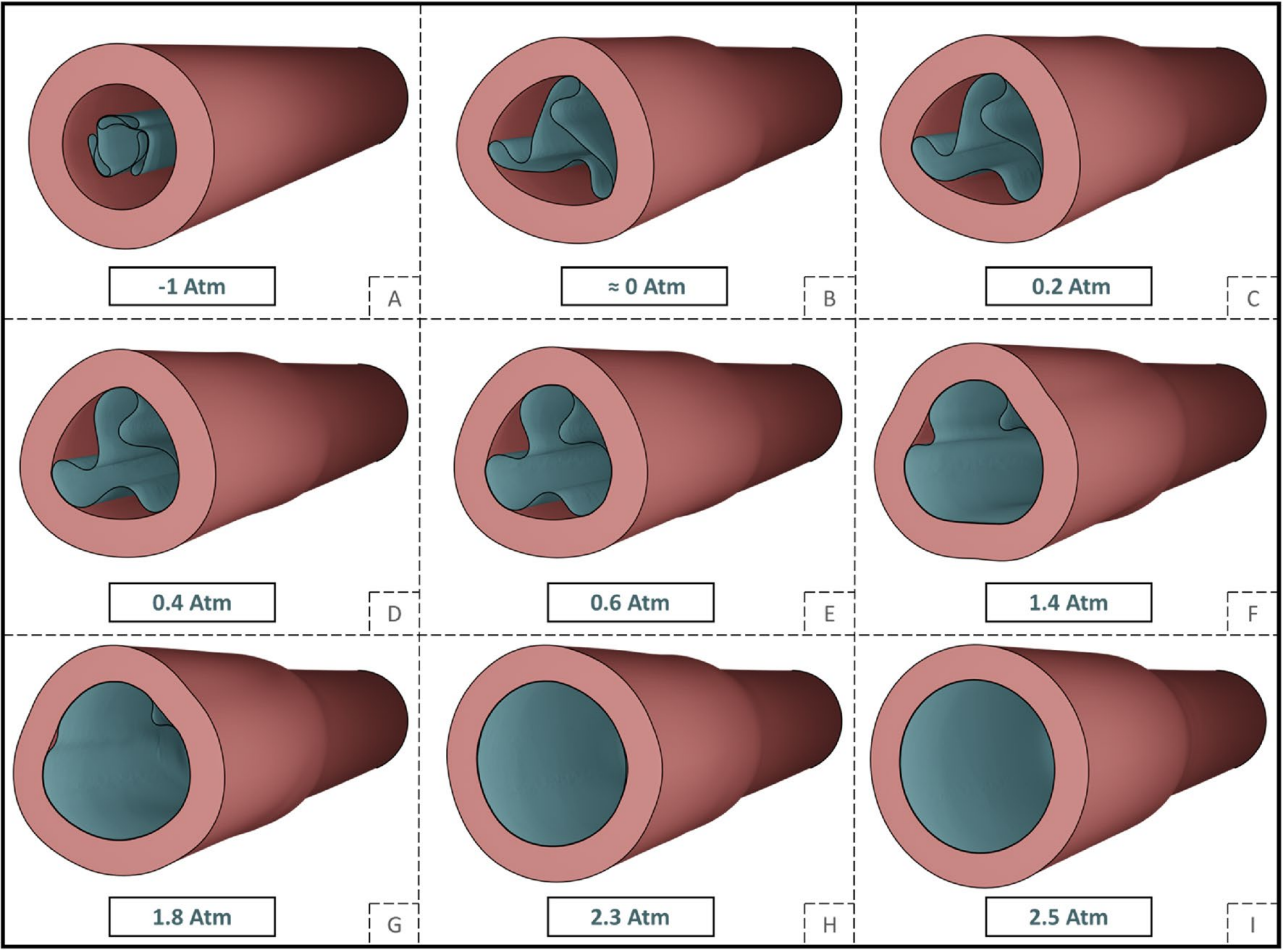
Early stages of the balloon expansion simulation along different expansion frames considering 40% DR. The figure unravels the different balloon-vessel configurations starting from the folded balloon (A) all the way until its complete distention (I). The presence of the vessel model increases the radial resistance of the system, resulting in a higher pressure required to distend the balloon compared to its free expansion process

In Fig. 3 (top panel), three curves of balloon diameter versus the Inflation Pressure (IP) are reported, corresponding to the balloon’s free expansion simulation for the three types of balloon wall thickness considered. The diameters at three key pressure levels are highlighted, as well as the circular configurations of the balloon at these pressures. For IP levels lower than the distention pressure, the maximum radial distance from the balloon’s axis was recorded at different frames and the corresponding values were calculated (Fig. 3, bottom panel). This affords the comprehension of the various balloon unfolding conditions and determines the configuration of the balloon during the initial contact with the vessel endoluminal wall. Consequently, the progression of the DCB deployment in the cylindrical vessel, with a 40% DR, reveals the various dynamic interactions between the balloon and the simulated vessel (Fig. 4). The initial interaction between the DCB and the vessel lumen occurs when the balloon is in a non-distended state, leading to an asymmetrical and irregular expansion pattern. The balloon’s folds apexes initially contact the vessel wall and continue to rotate, increasing the contact area as the IP increases.

**Fig. 5:**
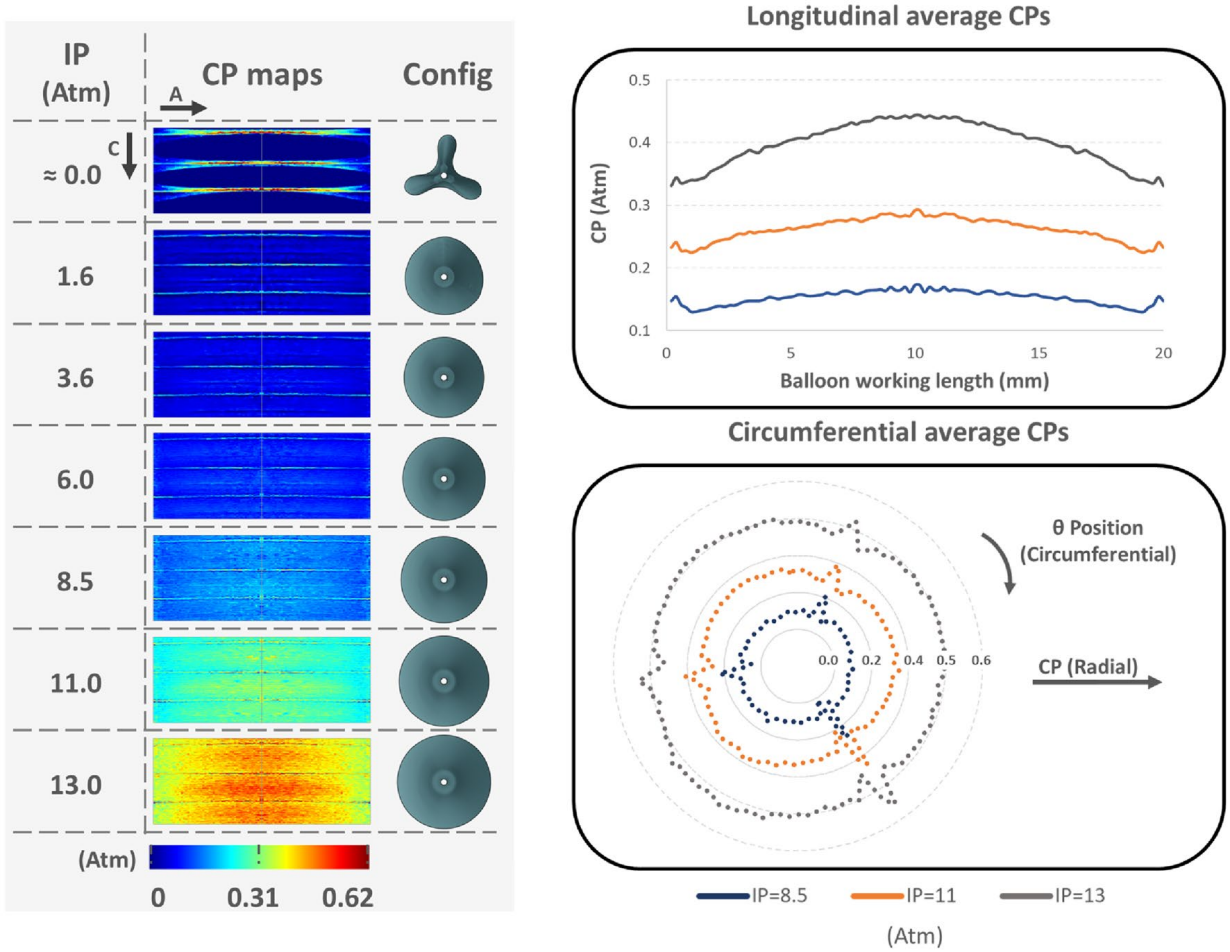
CP maps and the respective average values along the longitudinal and circumferential axis at different IPs for the control case simulation, while the last column depicts the concurrent configuration of the balloon. Linearly distributed imprints of the the folds are present even at high IPs, where the balloon has achieved its full distention. “A” and “C” on the top left of the figure denote the axial and circumferential direction of the artery respectively, while the right column shows the balloon’s configuration at the different IPs. A 10% variation of the wall thickness along the longitudinal axis of the balloon’s working length revealed a bell-shaped concentration of CP. This caused a circumferential non-uniformity of average CP

After analyzing the control case simulation, the two-dimensional CP maps of the vessel wall surface revealed non-uniform CP on the vessel endoluminal surface, both longitudinally and circumferentially (Fig. 5). At low IPs, linearly distributed CP concentrations developed where the folds touched the vessel; increasing the IP the balloon achieved a circular cross-section and fully engaged the luminal surface, the pressure globally increased but the linear concentrations were still present.

**Fig. 6:**
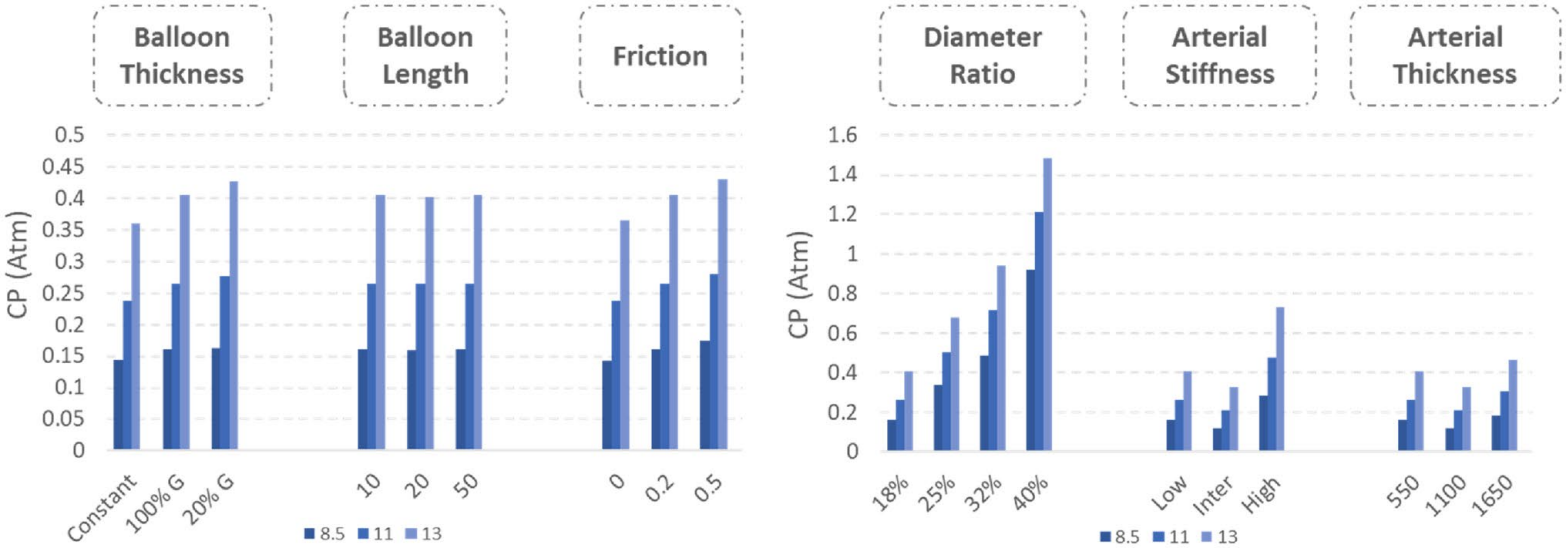
Global average CP values among different conditions for various IPs. The observed increase in the average values of CPs at different IPs can be attributed to the following changes: a decrease in balloon thickness, an increase in balloon length, an increase in the friction coefficient, an increase in the diameter ratio, an increase in arterial stiffness, and an increase in arterial thickness. Among these factors, the diameter ratio and arterial stiffness were found to have a more significant impact on the average CP

Average CP across the entire area of interaction between the vessel and the working length of the balloon was 10 to 60 times lower than the concurrent IP for the suite of simulations described in Table 1 (Fig. 6). 1.5-fold increase in the IP resulted in a roughly 1.5 to 2.5-fold increase in the CP values. The results also indicate that an increase in the friction coefficient at the balloon-vessel interface resulted in a slight increase in CP levels across all IPs, while the variation of the balloon length revealed no significant change in average CP values. In the balloon thickness sensitivity analysis, the Constant case revealed the lowest CP, whereas in the 20% Gradual case, the CP was the highest. Under conditions where vessel material stiffness was reduced or amplified, the average CP across the different IPs decreased by a factor of 0.43 to 0.46 and increased by a factor of 1.76 to 1.81, respectively. The alterations in vessel wall thickness showed similar behavior, but with a comparatively minor yet significant impact on the average CP: when the vessel wall thickness was halved compared to the control case, the developed CPs decreased by roughly 30%. The impact of DR on the resulting CPs along the vessel across four IPs was found to be the most significant. A steep increase in CP was observed upon increasing the IP from 8.5 to 13 atm. Increasing the DR from 18% to 40% led to a 2.6-to-4.7-fold increase in CP. When the IP increased by 50%, an 2.5-fold increase in CP was observed in the case of 18% DR, while the corresponding increase in the case of 40% DR was 1.6-fold.

**Fig. 7:**
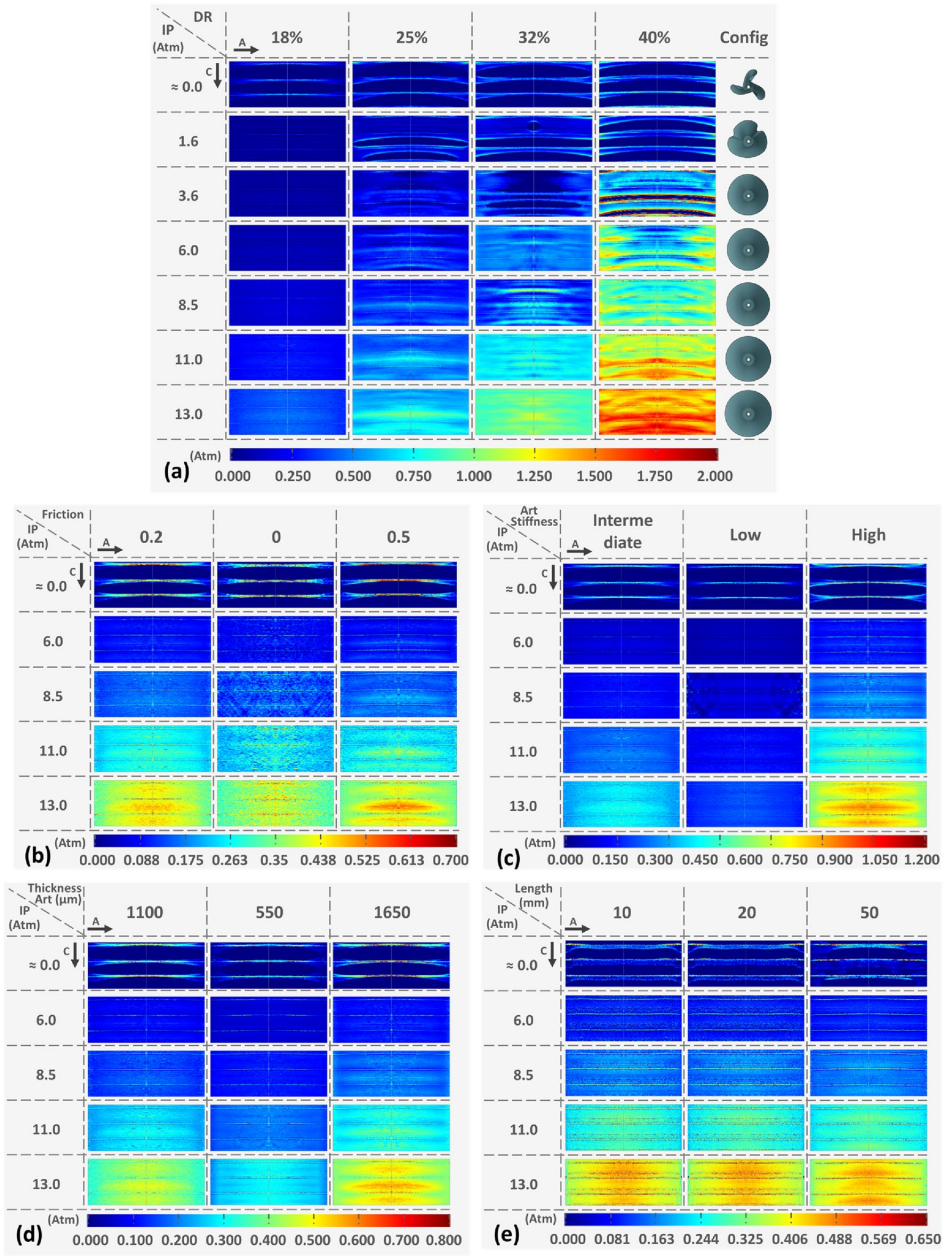
Color maps of different IPs with the respective developed CP maps on the vessel endoluminal area during the interaction with the DCB: **a** among the four different DR implemented (the last column represents the balloon configuration at the different IPs of the 40% DR case), **b** among the three different friction penalties considered, **c** among the three different vessel stiffness conditions assumed, **d** among the three different vessel thicknesses implemented in the simulations for different IP values, and **e** among the three different lengths implemented in the simulations for different IP values. The first column consistently represents the control condition, while alterations in CP amplitude produce distinct patterns of distribution. “A” and “C” on the top left of the figure denote the axial and circumferential direction of the vessel, respectively

The CP distribution color maps display linear patterns in all four cases of DR, which become increasingly pronounced as the level of DR increases, even at higher IP levels (Fig. 7a). The pressure distribution became highly irregular with the progression of DR conditions, while the initial contact mode determined the pressure distribution at later stages following the full inflation of the balloon. Low IPs exhibited areas of high CP concentration, similar to the globally high CP values at later stages of inflation.

Examination of the effect of three different friction coefficients on the distribution of CP maps reveals a progression of irregularity as friction coefficients increase (Fig. 7b). With a higher friction coefficient, a greater non-uniformity of contact is disclosed, manifesting as an increase by 30% in CP in one of the three folds. Sensitivity analysis in vessel stiffness revealed that the distribution of CP followed a systematic pattern in all the different IPs (Fig. 7c). Additionally, as the vessel stiffness increased, there were higher levels of CP in the central area of the balloons, with the linear patterned imprints of the folds becoming more prominent. Similarly, lower CP was observed in thinner vessels while thicker vessels were found to enhance contact non-uniformity (Fig. 7d). Different balloon lengths revealed no significant alteration on the CP distribution and magnitude (Fig. 7e).

**Fig. 8:**
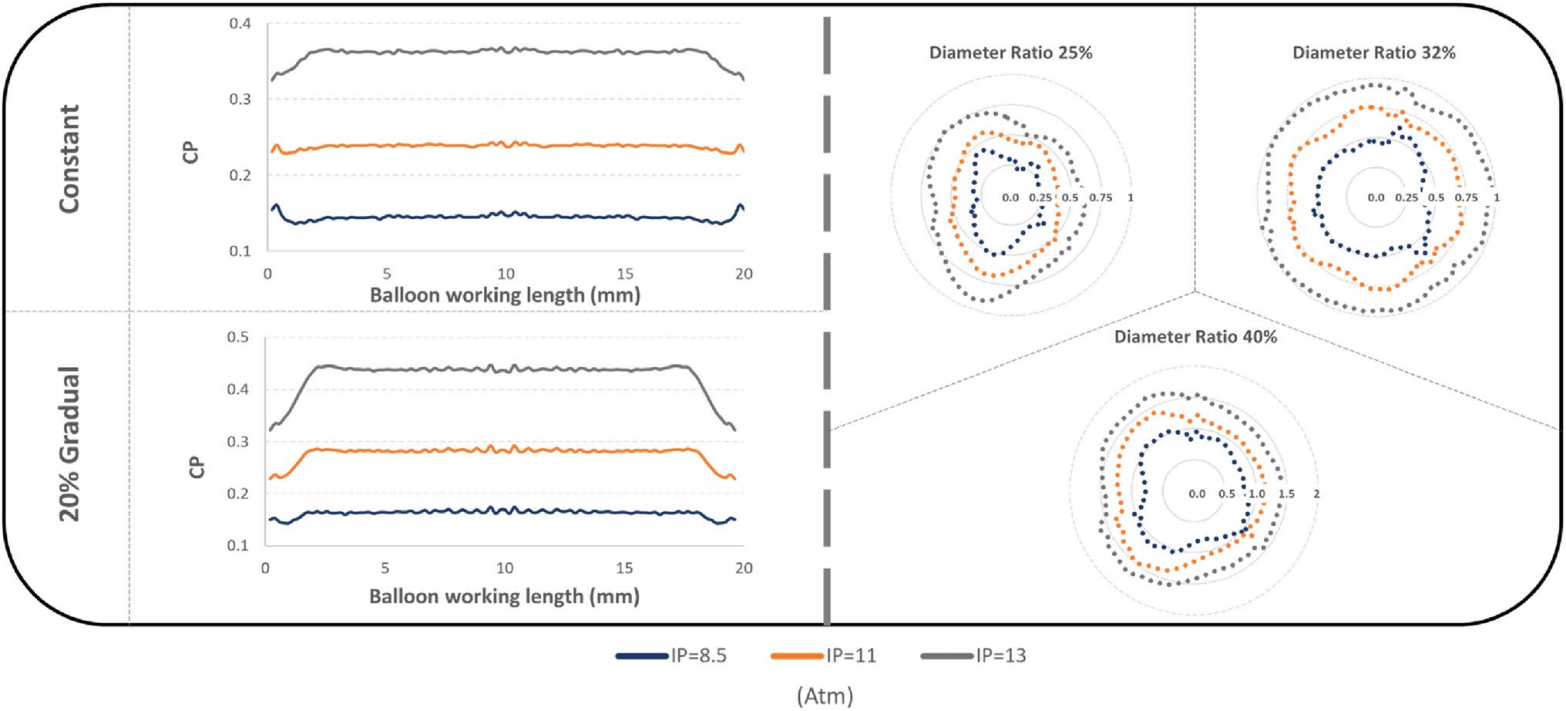
Left: Average CP values along the working length of the DCB among different numerically implemented thickness distributions at various IPs. CP was observed to be developed at low levels onto the endothelium located in the immediate vicinity of the proximal region of the balloon's working length. Moreover, the application of different degrees of balloon DR can lead to an eccentric distribution pattern of average CP across the vessel wall. Right: Average CPs along the circumferential axis of the vessel, among different IP values. The different blocks illustrate three of the DR conditions adopted in the simulations

By averaging the longitudinal rings of elements along the working length of the DCB (Fig. 8, left), it is determined that CP increases at the middle part of the balloon, an effect that becomes more evident as IP increases. Both the 20% Gradual and the Constant thickness cases display similar longitudinal variations, with the first exhibiting a 20% higher pressure at the central part of the balloon in comparison to the extremities, at 11 atmospheres of IP. Within the range of nominal IPs, the Constant thickness displays a uniform longitudinal distribution of CP in the central part of the working length and significantly reduced in the extremities. In contrast, the 100% Gradual condition manifests a greater degree of non-uniformity (Fig. 5).

Plotting of mean CP along the vessel circumference (Fig. 8, right), reveals the presence of an irregular distribution pattern that persists across all four DR conditions, while at elevated IP levels, the uniformity of CP appears to be re-established. The spatial distribution of CP exhibits an eccentricity at every stage of IP, while the pattern of contact is strongly influenced by the initial engagement of the folds with the vessel wall.

## Discussion

The objective of this study was to detect and characterize potential non-uniformities in contact patterns when an angioplasty balloon expands within an idealized vessel, with size and compliance resembling a femoral artery. A sensitivity analysis on various parameters revealed a repetitive trend of CP non-uniformity, with certain balloon features identified as key drivers. The results of the analyses further suggest CP as a driving force for coating transfer onto the arterial wall surface, and as a potential causative factor of non-uniform coating transfer in healthy pig arteries in vivo [56].

The folded configuration of the balloon was accounted to adequately describe the mechanical and geometrical response of the balloon and was consistent with with the experimental cross-section found by micro-CT [26]. During the unfolding process, the apex of the folds first makes contact with the luminal wall. This is explained by the star-shaped configuration during the initial stages of unfolding, before acquiring its cylindrical shape (Fig. 3). The subsequent interaction between the balloon and the vessel is dependent on the initial points of contact, and therefore, is also contingent upon the number of folds comprising the DCB. The CP of the vessel keeps track of the initial contact of the folds during the expansion process, influenced by the friction coefficient at the balloon-vessel interface. This phenomenon can be attributed to the balloon's tendency to rotate and recover its original cylindrical shape, which is impeded by friction. As a result, there are areas of concentrated stress that lead to an elevated interface pressure in those specific regions. The friction coefficient is a complex parameter that can be impacted by various factors such as the composition of the coating and vessel materials, their dynamic or static interaction, and the coating structure. The control friction coefficient was set at 0.2, since it was expected that the presence of coating on the balloon surface would increase this coefficient compared to values reported in a previous study [18]. Nevertheless, due to value uncertainty, this study simulated different friction coefficients and found that a higher friction coefficient results in irregular contact and the development of localized high CP values. Even after full balloon distension, some areas continue to exhibit higher CP (Fig. 5), particularly in cases of high DR, where the balloon's unfolding deviates from its free expansion process (Fig. 3). This effect is depicted in the corresponding CP maps (Fig. 7).

The balloon folding process resulted in linear patterned distributions of augmented CP concentration on the vessel mural surface. These findings are qualitatively consistent with images of in vivo transfer distribution on healthy pig arteries [56]. The correlation between the spatial distribution of CP and coating transfer, supports the hypothesis that the balloon unfolding process contributes to the aforementioned spatial coating distribution. In the same animal study, the mean longitudinal distribution coating imaged on the arterial surface was bell shaped with a peak centered in middle portion of the balloon’s working length. For the simulated case of 100% gradual thickness variation, averaging the CP on rings across the longitudinal axis of the vessel revealed significantly higher values in the middle area of the balloon and a slope similar to the in vivo longitudinal coating distribution [56].

Remarkably, even slight wall thickness variations across the working length of the balloon can result in significant variations in contact interaction. The results underscore the necessity of accurately modeling balloon membrane thickness in numerical studies of DCB angioplasty. Nevertheless, in all the thickness cases, the ends of the cylindrical area of the balloon, exhibited a decrease in the transfer of CP onto the mural surface.

As arterial stiffness and thickness vary with disease, we also investigated their impact on the CP developed during DCB angioplasty. As expected, the findings demonstrated that greater vessel stiffness and thickness were associated with a consistent rise in CP. This comes in agreement with the hypothesis that age-related arterial stiffening could lead to higher pressure during drug release [55] and, in turn, greater transfer of paclitaxel to the arterial wall [50]. In parallel, the non-uniform distribution of CP became increasingly pronounced both with increased vessel stiffness and thickness. Despite the higher CP levels observed in higher stiffness vessels, which usually correspond to diseased human peripheral vessels, the effectiveness of DCB angioplasty under these conditions remains controversial in the real case scenarios where patency decreases with calcification scores [20]. Moreover, preclinical studies using disease models have yielded inconclusive results. An in vivo analysis quantifying drug content in rabbit arterial vessels, after DCB treatment, revealed an increase in paclitaxel dose with increasing IP, in both healthy and diseased arteries. However, the findings suggest that the load of paclitaxel was lower in atherosclerotic arteries compared to healthy ones at low IP rates [52]. Contrastingly, another study using a different approach to induce atherosclerotic lesions identified a four-fold higher distribution of the drug in the diseased vessels in comparison to the healthy ones [21]. The study findings indicated that several factors may lead to uneven CP distribution during the interaction of a balloon with a cylindrical idealized vessel. Evidently, the geometrical irregularities and non-uniformities of patient-specific diseased vessels would increase the complexity of the interaction and the non-uniformity of the contact.

The current study had several limitations and certain assumptions were made in the numerical calculations. The adopted approach with respect to the parameter variation represents a limited exploration of the parameters space. However, the purpose of this sensitivity analysis was precisely to understand how the individual parameter under investigation can impact the simulation results in terms of contact pressure patterns and to observe whether the contact heterogeneity results were systematic. The obtained results, although representative of only a small portion of the entire parameter space, were considered sufficient for a semi-quantitative elucidation of the drivers of CP spatial patterns. The non-uniform thickness of the balloon was found to have a substantial impact on the distribution of CPs, however, the exact distribution along the length of the balloon was not fully described and the adopted thickness variations may not reflect real values. The non-uniform wall thickness of the balloon has also been implemented in stent expansion simulations and by applying an approximately 30% thickness variation from the distal to the proximal area along the longitudinal axis of the balloon resulted in a non-uniform stent deployment [47].

The balloon and vessel models considered in this study were modeled as single-layer, homogeneous, and isotropic materials with linear elastic and hyperelastic behavior, respectively. However, in reality, the balloon may exhibit an anisotropic non-linear response, although these effects may be negligible at low stretch values [25]. To accurately predict the CP maps on the balloon-vessel interface, during the balloon expansion, a more rigorous, validated modeling strategy should be adopted, able to capture the change of material properties induced by the folding process [26]. Nevertheless, the simplified approach adopted in the present study for modeling the mechanical properties of the balloon is considered adequate for the objectives of this work, namely, to investigate the CP patterns on idealized vessel. To this end, faithful numerical replication of the balloon expansion in vessels that replicated pig femoral arteries would require an anisotropic mechanical response of the arterial wall tissue [24, 31]. Nonetheless, the present study aimed to investigate spatial CP patterns rather than the absolute values of pressure, arising during the interaction of a rational, folded angioplasty balloon (Supplemental Fig. 2) with idealized vessels with a compliance comparable to that exhibited by peripheral arteries.

In essence, the objective of this study was to investigate the underlying causes of the patterns of coating transfer observed in animal studies [56]. This was accomplished through a series of simulations, wherein the contact pressure distributions consistently mirrored the qualitative patterns of coating transfer observed in vivo. The findings from this analysis offer valuable insights that can serve as a basis for future investigations. By incorporating the features described in this paper into a new study where a validated model of a real device, as well as a more sophisticated artery modeling are adopted, it becomes feasible to leverage numerical simulations to aid in the development of new guidelines for manufacturers and clinicians. Additionally, the results of this study offer potential avenues for investigating the relationship between the CP experienced by the interaction surface of the balloon and the efficacy of coating transfer. By utilizing experimental bench-top tests to compare different coating formulations, a correlation between the CP and coating transfer can be established [12, 23]. This can then inform numerical simulations of DCB angioplasty that incorporate patient-specific arterial geometry, allowing for a more realistic evaluation of the interaction conditions and the potential impact on coating transfer efficacy. Such integration of experimental and numerical approaches has the potential to enhance our understanding of the effectiveness of the DCB treatment and advance its clinical applications.

## Conclusion

In this study, an evaluation of the interplay between the vessel wall and the surface of an angioplasty balloon during its expansion was performed. The results indicated a significant irregularity of contact between the balloon and the vessel, affected by various conditions. More specifically, balloon to vessel ratio and vessel stiffness emerged as key parameters impacting the amplitude and distribution pattern of the developed CP. Furthermore, the thickness of the vessel wall exhibited a comparable influence, albeit with a lesser magnitude of impact, while the friction coefficient at the interface between the balloon and the vessel and the non-uniform wall thickness of the balloon may cause the concentration of the CP in certain regions of the vessel. Simulations identified the balloon’s folded configuration and its longitudinal thickness variation as major contributors to the observed irregularity of contact. This can explain the reduced and sporadic coating transfer to healthy porcine femoral arteries in vivo and ultimately result in the ineffectiveness of the treatment.

### Supplementary Information

Below is the link to the electronic supplementary material.

Supplementary file1 (PDF 596 kb)

## Abbreviations

DCB: Drug-coated balloon
CP: Contact pressure
IP: Inflation pressure
DR: Diameter ratio
