# Supplementary material for: Investigating Balloon-Vessel Contact Pressure Patterns in Angioplasty: In Silico Insights for Drug-Coated Balloons
Source: Ann Biomed Eng. 2023 Sep 26;51(12):2908–22. doi: 10.1007/s10439-023-03359-y (PMC10632265; doi:10.1007/s10439-023-03359-y)
Supplement: Supplementary file 1 — Supplementary file1 (PDF 596 kb) [file 10439_2023_3359_MOESM1_ESM.pdf]

Supplemental material

Mesh sensitivity

To ensure that the results of the numerical simulations were not influenced by the mesh used, a mesh sensitivity analysis was performed. In particular, the authors conducted a mesh refinement increasing systematically the number of elements in the circumferential direction of the balloon model during the simulation of the angioplasty balloon expansion into the vessel, applying an internal pressure ranging from 0 to 13 atm. The mesh sensitivity analysis targeted the elements around the circumference because the balloon is mainly subjected to circumferential and radial stress and strain during the inflation procedure. At each step of the refinement process, two factors were controlled at the maximum inflation pressure of 13 atm: i) the diameter of the lumen of the vessel (Supplemental Figure 1a) and ii) the contact pressure values as a mean of the values calculated at six critical nodes with increased contact pressure concentration (Supplemental Figure 1b). The initial mesh consisted of 220 shell elements along the circumference and 100 along the working length of the balloon. The authors added 20 elements circumferentially during each iteration.

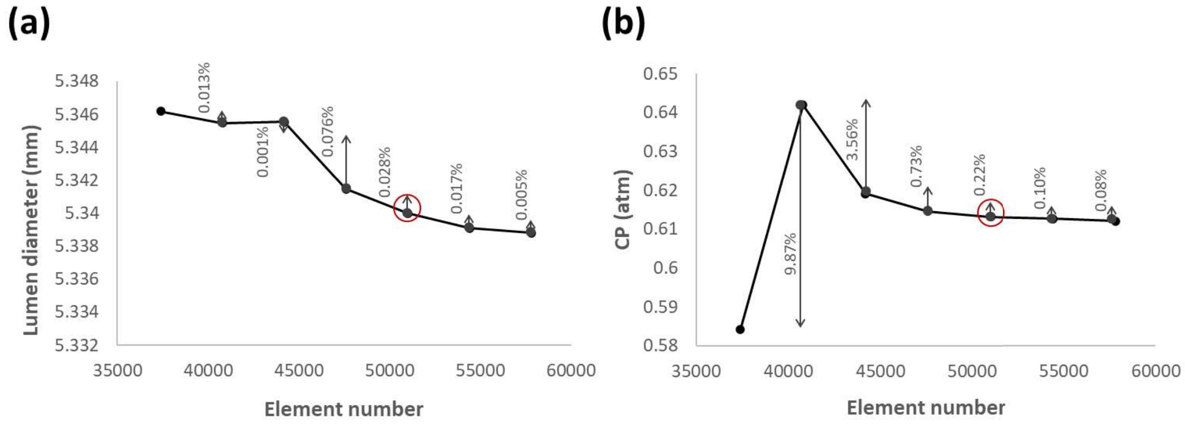

Supplemental Figure 1. Results of the mesh sensitivity analysis: (a) Diameter of the vessel lumen at the maximum inflation pressure of 13 atm: the percentile differences with each increase in element number are also shown. The red circle highlights the selected mesh. (b) Mean contact pressure between the balloon and the vessel among the six critical nodes at the maximum inflation pressure of 13 atm: the percentile differences with each increase in element number are also shown. The red circle highlights the selected mesh.

The simulations run on a 125 Gb RAM with 32 domains and domain parallelization of 32 CPUs. Through these simulations, the values of CP and lumen diameter converged adequately when they reached a mesh with 300 circumferential elements, balancing the accuracy and the computational time needed.

Validation

In the absence of physical balloon specimens, the authors of this study made use of a generic drug-coated balloon as a reference point. Consequently, in order to evaluate the model's reliability in emulating the folded configuration, a comparison was conducted between the cross-sectional characteristics of the digital device and an actual physical device. This comparative analysis adhered to the methodology introduced in the research conducted by Geith et al. [1], and the physical balloon's folded configuration depicted in the same study was used in the present study as a reference. As depicted in Supplemental Figure 2, a favorable concordance was observed between the experimental folded configuration (quantified through micro-CT scanning and documented in Geith et al.[1]) and the numerical results obtained in this current study.

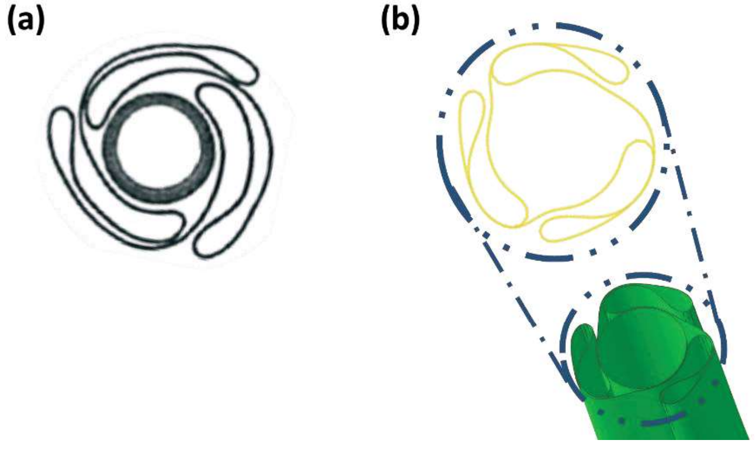

Supplemental Figure 2. Validation of the folded balloon configuration: (a) Cross section of the folded balloon employed in Geith et al. [1], acquired from micro-CT images, and (b) the cross-section of the balloon's numerical model after performing the folding process in this current study.

References

1. Geith, M. A., K. Swidergal, B. Hochholdinger, T. G. Schratzenstaller, M. Wagner, and G. A. Holzapfel. On the importance of modeling balloon folding, pleating, and stent crimping: An FE study comparing experimental inflation tests. *International Journal for Numerical Methods in Biomedical Engineering* 35:e3249, 2019.
